# Faecal egg count reduction tests and nemabiome analysis reveal high frequency of multi-resistant parasites on sheep farms in north-east Germany involving multiple strongyle parasite species

**DOI:** 10.1016/j.ijpddr.2024.100547

**Published:** 2024-05-05

**Authors:** Jürgen Krücken, Paula Ehnert, Stefan Fiedler, Fabian Horn, Christina S. Helm, Sabrina Ramünke, Tanja Bartmann, Alexandra Kahl, Ann Neubert, Wiebke Weiher, Ricarda Daher, Werner Terhalle, Alexandra Klabunde-Negatsch, Stephan Steuber, Georg von Samson-Himmelstjerna

**Affiliations:** a Institute for Parasitology and Tropical Veterinary Medicine, Freie Universität Berlin, Germany; b Veterinary Centre for Resistance Research, Freie Universität Berlin, Berlin, Germany; c Federal Office of Consumer Protection and Food Safety, Berlin, Germany

## Abstract

Anthelmintic resistance in sheep parasitic gastrointestinal nematodes is widespread and a severe health and economic issue but prevalence of resistance and involved parasite species are unknown in Germany. Here, the faecal egg count reduction test (FECRT) was performed on eight farms using fenbendazole, ivermectin and moxidectin and on four farms using only moxidectin. A questionnaire was used to obtain data on management practices to potentially identify risk factors for presence of resistance. All requirements of the recently revised WAAVP guideline for diagnosing anthelmintic resistance using the FECRT were applied. Nematode species composition in pre- and post-treatment samples was analysed with the nemabiome approach. Using the eggCounts statistic package, resistance against fenbendazole, ivermectin and moxidectin was found on 7/8, 8/8 and 8/12 farms, respectively. No formal risk factor analysis was conducted since resistance was present on most farms. Comparison with the bayescount R package results revealed substantial agreement between methods (Cohen's κ = 0.774). In contrast, interpretation of data comparing revised and original WAAVP guidelines resulted in moderate agreement (Cohen's κ = 0.444). The FECR for moxidectin was significantly higher than for ivermectin and fenbendazole. Nemabiome data identified 4 to 12 species in pre-treatment samples and treatments caused a small but significant decrease in species diversity (inverse Simpson index). Non-metric multidimensional scaling and k-means clustering were used to identify common patterns in pre- and post-treatment samples. However, post-treatment samples were scattered among the pre-treatment samples. Resistant parasite species differed between farms. In conclusion, the revised FECRT guideline allows robust detection of anthelmintic resistance. Resistance was widespread and involved multiple parasite species. Resistance against both drug classes on the same farm was common. Further studies including additional drugs (levamisole, monepantel, closantel) should combine sensitive FECRTs with nemabiome data to comprehensively characterise the anthelmintic susceptibility status of sheep nematodes in Germany.

## Graphical abstract

**Figure:**
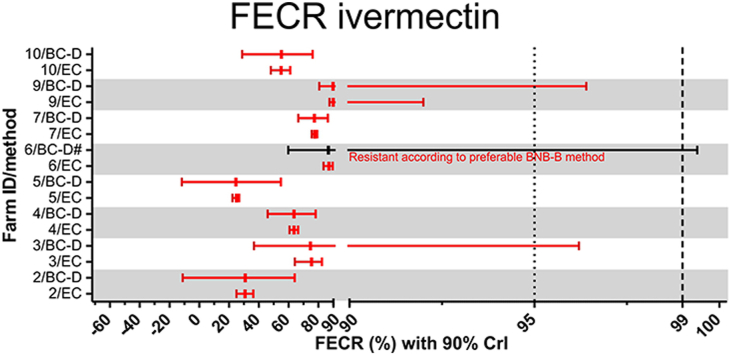

## 1 Introduction

Infections with gastrointestinal nematodes can cause severe animal welfare problems in small ruminants and lead to e.g. decreased weight gain, anaemia, diarrhoea, and oedema (Dargie, 1980). The most frequently used control measures have been metaphylactic treatments with anthelmintic drugs for the last decades (Claerebout et al., 2020; Zajac and Garza, 2020). Broad-spectrum nematocidal drugs commonly used nowadays in small ruminants include the benzimidazoles (BZs) (e.g. fenbendazole (FBZ), albendazole), the macrocyclic lactones (MLs)(e.g. ivermectin (IVM), moxidectin (MOX) and doramectin), and the imidazothiazole levamisole (Selzer and Epe, 2021). The spiroindole derquantel is available as combination with the ML abamectin in many countries (Gilleard et al., 2021) but not in the European Union. In addition, some anthelmintics with a narrower spectrum of target species are on the market such as monepantel (aminoacetonitrile derivative active against strongyle nematodes) (Sager et al., 2009a) and closantel (salicylic acid derivative with specific activity against blood-feeding nematodes such as *Haemonchus contortus*) (Swan, 1999).

Anthelmintic resistance (AR) is a widespread problem in gastrointestinal nematodes of small ruminants, cattle and horses (Charlier et al., 2022; Nielsen, 2022), while the situation in companion animals shows only a limited problem with AR (von Samson-Himmelstjerna et al., 2021) although multi-drug resistant canine hookworms are becoming an increasingly severe problem in the USA (Jimenez Castro et al., 2021, 2022; Venkatesan et al., 2023). Resistance against all new drug classes has evolved within a few years after initial introduction into the pharmaceutical market. In small ruminants, the situation is probably worst with widespread resistance as well as multi-drug resistance in countries in the global south such as Australia, New Zealand, South Africa and many countries in South America (Gilleard et al., 2021).

In the field, anthelmintic resistance is most typically diagnosed using the faecal egg count reduction test (FECRT) (Kaplan et al., 2023). This test has the advantage that it can be applied to test in vivo the anthelmintic susceptibility of most parasite species. Furthermore, it is relatively simple to perform and it does not require any sophisticated equipment. One of its major disadvantages is its limited sensitivity (Königová et al., 2021). It was shown that the FECRT was not able to detect resistance although roughly 25% of a nematode community was resistant (Martin et al., 1989). Other disadvantages are that the test is very time-consuming and labour-intensive, which also makes it rather expensive. For a long time, the FECRT was conducted according to a WAAVP guideline published by Coles et al. (1992) but this guideline was only recently revised considerably (Kaplan et al., 2023). In comparison to the original guideline, the revised guideline contains multiple changes: First, it strongly recommends a paired study design using pre- and post-treatment egg counts instead of using an untreated control group. Second, instead of a minimum mean egg per gram (epg) pre-treatment the revised guideline proposes to use a minimum number of eggs counted under the microscope (raw egg count of 200). Third, the revised guideline specifies new, host species-specific target thresholds for anthelmintic efficacy, for sheep this corresponds to 99% target efficacy and a grey zone of 95%–99% in the revised vs. 95% target efficacy and a grey zone of 90%–95% in the original guideline. Fourth, the efficacy is interpreted using the upper and lower 90% confidence/credible limits and not the estimate for the FECR and its lower 95% confidence/credible limit.

In order to identify the nematode species involved in resistance and multi-drug resistance, an approach is necessary that can be applied to all strongyle nematode species and allows the responses to all drugs. A wide range of conventional PCR, real-time PCR and deep-amplicon-sequencing approaches have been described for this purpose (Rinaldi et al., 2022). Many of the methods only allow the identification of the species present in pre- and post-treatment samples. However, quantification of the species composition and its comparison between pre-and post-treatment samples is of course far more informative. Semi-quantitative approaches have been developed that allow to identify and roughly quantify the major strongyle nematode species in a sheep or cattle faecal sample (Roeber et al., 2012a, 2012b). Individual real-time PCRs for each strongyle species need to be conducted. Thus, an initial knowledge about the species spectrum that can be expected on a farm is required and the quality of this “informed guess” might have a strong impact in cases where unexpected species significantly contribute to the parasite burden.

The nemabiome approach (Avramenko et al., 2015; Baltrušis et al., 2022) is the most advanced option for the analysis of the gastrointestinal nematode species composition available today and allows a more unbiased view on the strongyle nematode community in a sample. The method is based on a pan-strongyle internal-transcribed-spacer-2 (ITS-2) PCR that has been applied to a very high number of strongyle species since its first characterization by (Gasser et al., 1993) and is one of the most frequently used tools for molecular species identification in this parasite group. This has the advantage that for a large number of species and virtually all economically relevant species a reference sequence is available in sequence databases such as NCBI GenBank. A disadvantage is that the ribosomal gene cassette including the ITS-2 is a multi-copy gene and the copy number varies between species which complicates relative quantification and comparison of parasite stages between samples using number of reads in a sample in the following deep-amplicon-sequencing step on an Illumina MiSeq. This is further complicated by the fact that the number of cells in a certain stage is also unevenly distributed and differs between species. First (L1) or third (L3) stage larva of *H. contortus* do not necessarily contain exactly the same number of cells as the corresponding stages of *T. circumcincta*. For this purpose, correction factors have been established to counteract these effects and calculate frequency of parasite stages from frequency of reads mapping to the reference sequence for this species in a database. Another disadvantage might be that between very closely related species the number of polymorphisms in the ITS-2 is limited and in this case the discriminatory power might be suboptimal (Ramünke et al., 2018).

The study design was according to recommendations made by the COMBAR group (https://www.combar-ca.eu/, last visited 16. December 2023) for the FECRT that largely relied on suggestions made by (Kaplan, 2020). These suggestions made the recommendations of the WAAVP guideline available before the guideline was published in 2023 and are completely in line with the recommendations in the revised WAAVP guideline for the conduction of the FECRT (Kaplan et al., 2023). The present study aimed to (i) obtain initial data regarding the anthelmintic susceptibility status of ovine strongyle populations in north-east Germany, (ii) evaluate the effects of the revised WAAVP guideline for the FECRT on the interpretation of the results compared to the criteria of the original guideline, (iii) identify the parasite species involved in resistance and multi-drug resistance and (iv) determine the species α and β diversity of ovine strongyle parasites in the region and the effect of treatment on diversity.

## 2 Materials and methods

### 2.1 Recruitment of farms

Farms were not randomly selected but identified based on the willingness of the farmers to participate. Moreover, it was required that it was possible to capture the animals twice for sampling and treatment, that animals had not been treated against parasitic nematodes for at least six weeks and that at least 15 sheep with an age below 24 months were available. The study was announced in the journal of the local sheep and goat breeder's organization (Schafzuchtverband Berlin Brandenburg e.V.) and members of the organization were also directly contacted and asked for their interest to participate.

### 2.2 Sample collection and treatment

Farms were visited between September and November 2020. Up to 75 animals were included on the farms. If enough animals were available, sheep were randomly assigned to three groups (15–40 sheep/group) and the groups were assigned to treatment with either FBZ, IVM or MOX. On some small farms, only efficacy of MOX was tested, since it was expected that resistance against this drug might be less advanced than for FBZ and IVM. Faecal samples were collected from the rectal ampulla and stored in the examination glove. Animals were treated with FBZ (Panacur suspension 2.5% [Intervet Deutschland], 5 mg/kg body weight orally), IVM (Alfamectin 1% injectable [alfavet Tierarzneimittel], 200 μg/kg body weight subcutaneously), MOX (Cydectin 0.1% [Zoetis Deutschland], 200 μg/kg body weight, orally) or 2.5 mg/kg monepantel (MON) (Zolvix 25 mg/kg [Elanco Deutschland]). In order to apply the correct dose, all animals were weighed on an animal scale (Nohlex GmbH, Buchholz, Germany) or a scale provided by the farmer. A second sample was collected from the same animals usually 14 days post-treatment. However, due to limitations in the availability of the farmers, on some farms the second visit was 13–16 days post-treatment. Animal handling was in accordance with European (European Directive, 2010/63/EU) and German (German Animal Welfare Act [Tierschutzgesetz]) laws. The study design was presented to the Landesamt für Arbeitsschutz, Verbraucherschutz und Gesundheit (LAVG) Brandenburg as the responsible administrative state authority for animal experiments. The authority decided that the procedures are not an animal experiment and do not require a formal permission (AZ-2340-9-2020).

### 2.3 Questionnaires

A questionnaire was used to obtain data about management of the farms to be used for statistical analyses. Data were collected for instance regarding: Contact details, sheep breeds kept, other animal species kept on the holding, the total number of sheep with a breakdown into adult ewes, rams, young ewes (1–2 years) and lambs (<1 year), the main types of use (meat or milk production, landscape preservation), whether sheep breeding was a main or secondary business, the average number of animal acquisitions per year and the origin of the animals, quarantine and treatment procedures for newly introduced sheep, the type of husbandry in which the animals were kept, location and size of the pasture area(s), duration of animal housing during the winter months, access to forest edge or water bodies, re-grazing of the animals on cattle pastures, the type of watering of the animals on the pastures, use of anthelmintics on the farm (Were certain anthelmintics used on the farm in the past and why?), the frequency of anthelmintic use in different age groups, whether simultaneous deworming of all animals per treatment group was performed or if animals were dewormed based on certain criteria, the use of individual anthelmintic products, the person responsible for deworming (veterinarian vs. shepherd), the use of coproscopical controls to diagnose worm infections and to determine the success of treatment on the farm. Lastly, the farmer's personal self-assessment of the deworming regime applied was determined using questions with a grading system (Likert scores with four levels). In this section, the following questions were asked: Do you feel sufficiently well advised by your vet/animal health service on deworming strategies? How confident are you in making decisions about deworming your sheep? Do you feel well informed about deworming? Would you like more information on deworming? Do you think regular faecal diagnosis on your farm is useful?

### 2.4 Faecal egg count determination

Faecal egg counts were determined with a modification of the Mini-FLOTAC method without using the Fill-FLOTAC device as detailed recently (Boelow et al., 2022). In brief, 5 g of faeces were weight on a scale, homogenized in 45 ml saturated NaCl solution (relative weight 1.2), filtered through a sieve (about 0.5 mm mesh size) and the flow through was used to fill the Mini-FLOTAC chambers. For each sample, two counting chambers in a Mini-FLOTAC device were used resulting in a multiplication factor of 5 to calculate the epg from the raw egg counts. For farm 10, the total number of eggs counted for all animals was below 200 (raw egg counts), which was considered to be the minimum to obtain a good power (Kaplan, 2020; Kaplan et al., 2023). For this farm, all samples were analysed with the same method a second time and the number of observed eggs in the four Mini-FLOTAC chambers for each animal was multiplied with 2.5 to obtain the epg. After two rounds of Mini-FLOTAC examination, the raw egg count was above 200 for all farms.

### 2.5 Egg isolation and preparation of first stage larvae

For nemabiome analyses of sheep samples, the correction factors to calculate frequency of parasites from frequency of reads have been established based on L1 for sheep parasitic nematodes (Redman et al., 2019). Therefore, eggs were purified from faecal samples pooled on the farm level and post treatment also individually for each treatment group. For this purpose, all available faecal matter that was not used for the Mini-FLOTAC procedure was used. Egg purification was done on the same day as samples were collected in the field. Faecal matter was suspended in tap water and filtered through a sieve with mesh size with >250 μm. The suspension was filled into multiple 80 ml centrifugation tubes (Duran, glas) and centrifuged at 1500×*g* for 5 min. The supernatant was removed and additional suspension was loaded on top of the pellets and re-centrifuged until the material for the whole suspension was pelleted. Pellets were merged and resuspended in saturated NaCl solution (one volume of sediment plus 3 vol of salt solution) and again centrifuged at 1.500×*g* for 5 min. The top 5–10 ml of the supernatant were transferred to a new tube that was filled with tap water using 4 vol of tap water for a volume of salt solution. After centrifugation at 1500×*g* for 5 min, supernatants were removed, pellets were merged and the eggs were resuspended in 10 ml water (mixture of distilled water and tap water with an electric conductivity of 250 μS). Depending on the number of eggs, this suspension was either immediately used or further purified on a sucrose step gradient, especially for pre-treatment samples, for which the number of available eggs was generally higher, to remove contaminating faecal material. A sucrose stock solution was prepared by dissolving 60 g sucrose in 40 ml distilled water. The solutions for the step gradient contained 10%, 25% and 40% of the stock solution diluted with distilled water. Solutions were stained with different food colours to easily identify the borders between different densities. The step gradient was overlaid with the egg suspension and the gradients were centrifuged at 1500×*g* for 5 min without brake. The eggs were visible as a white veil between the 10% and 25% layers and were collected with a Pasteur pipette. They were washed on a 25 μm sieve with tap water to remove the sugar solution and eggs were collected in a fresh tube and were centrifuged at 1500×*g* for 5 min. Supernatants were removed and the eggs were resuspended in 10 ml water (electric conductivity of 250 μS).

Purified eggs were transferred to a T-25 cell culture bottle for suspension cells and counted by transferring three 10 μl aliquots on a microscope slide and counting the number of eggs under a microscope. Eggs were incubated at 25 °C for 48 h. After L1 had hatched, the content of the cell culture flask was transferred to a 15 ml centrifuge tube and rinsed with 5 ml DEPC-treated water and left for 30 min. The supernatant was removed to 5 ml and the L1 were counted and aliquots of at least 1000 larvae were frozen in a total volume of 300 μl at −20 °C.

### 2.6 DNA isolation, internal-transcribed-spacer-2 PCRs and deep amplicon sequencing

DNA was isolated from frozen L1 using the Macherey-Nagel SOIL-Kit we have frequently used for isolation of DNA from faecal samples before (Demeler et al., 2013; Krücken et al., 2017), which includes a step to remove PCR inhibitors from samples. The kit contains a tube with beads for sample homogenisation that was performed using a SpeedMill (Jena Bioscience) using four homogenisation cycles for 60 s interrupted by 60 s without shaking. DNA was purified from the homogenate using the recommendations of the manufacturer and eluted with 50 μl dH_2_O (PCR grade).

The first PCR was conducted at FUB using modified NC1/NC2 primers that contained Illumina adapter sequences. Between the NC1/NC2 sequences and the Illumina adapters, 0-3 additional random bases (N) were inserted (see (Avramenko et al., 2015) for details and Table S1). This strategy avoids that the same colour signal is detected at all positions of the Illumina flow cell during initial sequencing of the primer, which could lead to a low diversity library. This must be avoided to enable the correct calculation of correction factors.

For library preparation, PCR products were transferred to the NGS laboratory of the BVL. The resulting PCR products were cleaned using AmpureXP beads (Beckman Coulter GmbH, Krefeld, Germany) according to the manufacturer's protocol with a final elution volume of 40 μl 10 mM Tris-HCl buffer (pH 8.0) to remove primer dimers and protein residues. The DNA was then quantified using the Qubit dsDNA HS Assay Kit (Thermo Fisher Scientific, Darmstadt, Germany) on a Qubit 4 Fluorometer (Thermo Fisher Scientific, Darmstadt, Germany). To add Illumina adapters, 10–20 ng of purified PCR product was further used. The index PCR was carried out using the IDT for Illumina DNA/RNA UD Indexes Set (Illumina, San Diego, CA, USA) and the KAPA HiFi HotStart Ready Mix (Roche Molecular Systems, Pleasanton, CA, USA). Purified PCR product (3 μl) was mixed with 12.5 μl of Kapa HiFi Ready Mix, 1.5 μl of dual index primer and 8 μl of PCR grade water. The amplification conditions using the Kapa HiFi Ready Mix in a final reaction volume of 25 μl were as follows: initial denaturation at 98 °C for 45 s, followed by 7 cycles of denaturation for 20 s at 98 °C, annealing for 20 s at 63 °C, elongation for 20 s at 72 °C and a final extension for 2 min at 72 °C. After the index PCR, the PCR products were cleaned again using AmpureXP beads according to the manufacturer's protocol with an elution volume of 25 μl 10 mM Tris-HCl buffer. The final libraries were quantified before pooling using the Qubit dsDNA HS Assay Kit and diluted to 4 nM in 10 mM Tris-HCl buffer. The diluted libraries were pooled in equimolar amounts, denatured and finally diluted according to the manufacturer's protocol (Illumina). The sample pool was sequenced on a MiSeq benchtop sequencer (V3, 2 × 300 bp, Illumina). At least 20,000 paired end reads should be generated for each sample.

### 2.7 Statistical analyses

#### 2.7.1 Calculation of faecal egg count reduction with credible intervals

Since on some farms the multiplication factor of 2.5 produced epg values that did not correspond to natural numbers, all analyses to estimate the FECR were based on raw egg counts before multiplication. The eggCounts package 2.3-2 (Paul R Torgerson et al., 2014; Wang et al., 2018) was used in R 4.1.3 to obtain the estimate for the FECR and its 90% and 95% credible intervals (CrIs). For this purpose, eggCounts uses a Bayesian approach and estimates these parameters using a Markov chain Monte Carlo approach. The eggCounts algorithm was used with paired data (pre and post-treatment from the same animal) but without individual efficacy of the drug (farm wide identical efficacy) and without zero inflation. All other parameters and priors were used according to the default settings. As estimate of the FECR, the mode of the posterior density distribution was used and as 95% CrI the 95% highest posterior density interval (HPD) as recommended by (Wang et al., 2018). In order to calculate the 90% CrI, the “stan” object in the output of eggCounts was converted to a “mcmc” object using the stan2mcmc function of eggCounts. This “mcmc” object was further analysed using the HPD interval function from the coda 0.19–4 package to extract the values of the 90% HPD interval to be used as 90% CrI. In addition, data were analysed with the bayescount R package 1.1.0 using the web-based portal www.fecrt.com (last visited 16. December 2023) and a paired data model was chosen. Since the bayescount package does not provide an estimate of the FECR but only of the 90% CrI, the average reduction in FEC was used.

In order to compare the interpretation of the results obtained with both packages, farms were categorised as resistant (including low resistant for which the lower 90% confidence/credible limit is above 95%), inconclusive (or suspected resistance) and susceptible and the inter-rater agreement between eggCounts and bayescount was calculated as Cohen's κ coefficient using the CohenKappa function from DescTools package 0.99.48. For the eggCounts results, a further comparison was computed: The interpretation based on FECRT and lower 95% credibility/confidence limit as proposed in the original WAAVP guideline on the FECRT (Coles et al., 1992) was compared with the new interpretation based on upper and lower 90% CrI as proposed recently by (Kaplan et al., 2023). Cohen's κ values were interpreted according to (Landis and Koch, 1977).

The 95% confidence intervals (CIs) for frequencies of resistance to different drugs were calculated using the binom.wilson() function from the epitools package 0.5–10.1 in R 4.1.1. Pairwise comparison of frequencies between groups was conducted using the mid-p exact test as implemented in the tab2by2() function from the same package.

#### 2.7.2 Assignment of reads to species and calculation of species frequencies

Sequence reads from the nemabiome analysis were demultiplexed and adapters as well as primers were removed using cutadapt. The dada2 pipeline (Callahan et al., 2016) was used for further analysis as detailed on the nemabiome web page (https://www.nemabiome.ca/; last visited 16. December 2023). Reads were filtered and trimmed to include only reads with a maximum expected error of 2 for the forward and five for the reverse reads and reads were truncated after a quality score of at maximum two expected errors per read. After training dada2 on the error profile of the actual dataset, error-correction (denoising) was performed. Then, read pairs were merged into a single sequence and chimeric sequences were removed. IdTaxa from the DECIPHER 2.22.0 package was used to assign sequences to taxa using a threshold of 60% using 100 bootstrap replicates. For this purpose, version 1.3 of the nemabiome ITS-2 database was used (Workentine et al., 2020).

Species diversity was calculated as inverse Simpson index for each farm/treatment group using the diversity() function from the vegan package 2.6–4. Species diversities between samples from untreated and treated animals were compared using the Mann Whitney *U* test in GraphPad Prism 5.03. The function iNEXT() from the iNEXT 3.0.0 package was used to estimate species richness (Chao1 index) on each farm/treatment group level.

#### 2.7.3 Non-metric multidimensional scaling and cluster analyses

In order to identify potential common treatment effects and patterns in species composition before and after treatment, non-metric multidimensional scaling (NMDS) and cluster analyses were used. For both analyses, read numbers after applying the species-specific correction factors were used and these were transformed to relative frequencies (in percent). Using the vegan package 2.6–4 (Oksanen et al., 2022), a dissimilarity matrix was calculated based on relative frequencies using the vegdist() function applying a Bray-Curtis dissimilarity transformation. Non-metric multidimensional scaling was conducted using the metaMDS() function and the result was plotted using ordiplot(). The adonis2() function was used to evaluate potential differences between treatment groups using a permutation test. For this purpose, two different approaches were used, first testing untreated vs. treated samples and second testing untreated vs. FBZ, IVM and MOX treated samples. For hierarchical clustering, the hclust() function from the R base stats package was applied on the Bray-Curtis dissimilarity matrix. The results of all available clustering methods were evaluated. In addition, k means clustering was conducted using NbClust (NbClust package 3.0.1) applying all available indices on the dissimilarity matrix.

## 3 Results

### 3.1 Included farms

In total, twelve farms were recruited for the study, eleven from the federal state of Brandenburg and one from Mecklenburg-Western Pomerania, the state immediately north of Brandenburg. On eight of the farms, all three drugs (FBZ, IVM and MOX) were evaluated while on four farms only MOX was tested. Two farms, both with resistance against multiple drugs, were asked if a second treatment with MON could be performed but only one of the farmers agreed to participate in this part of the study.

### 3.2 Faecal egg count reduction test results

#### 3.2.1 Faecal egg count reductions and interpretation of the results

**Fig. 1:**
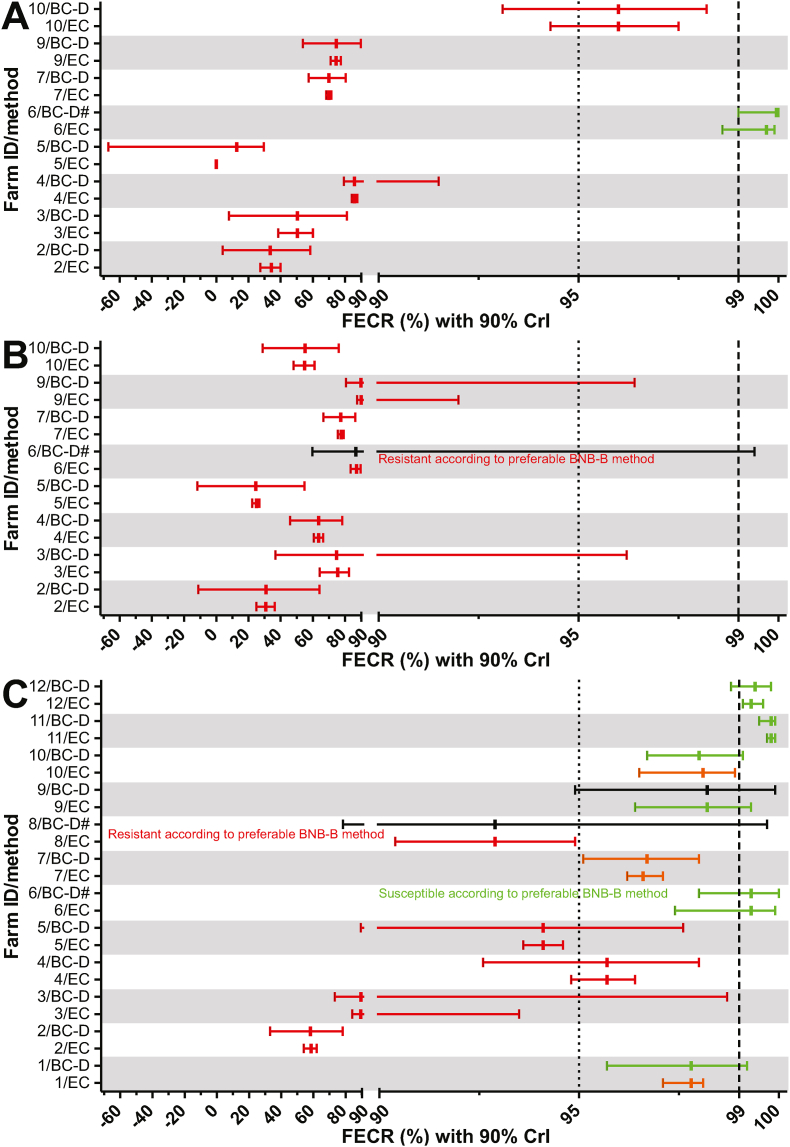
Faecal egg count reduction (FECR) estimates for strongyle parasites with 90% credible intervals (CrIs) for fenbendazole (A), ivermectin (B) and moxidectin (C). Using the eggCounts (EC) and bayescount (delta method, BC-D) R packages, the 90% CrIs were calculated. While the eggCounts package also provides an estimate for the FECR, the bayescount package only provides the CrI. Therefore, the FECR was calculated simply from mean pre and post egg counts. For sheep, the grey zone is located between 95% and 99% FECR and indicated by vertical lines. Colours indicate susceptible (green), resistant (red), low resistant (orange) and inconclusive (black) results. If the delta method was not the preferred method according to the bayescount approach (indicated by a #), the result of the BNB-B method is provided in a comment. (For interpretation of the references to colour in this figure legend, the reader is referred to the Web version of this article.)

**Fig. 2:**
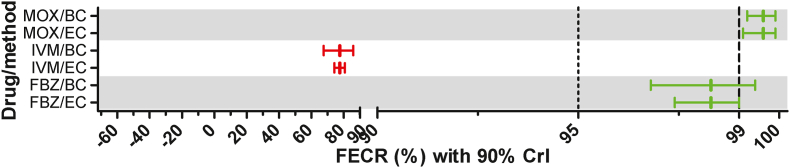
Faecal egg count reduction (FECR) estimates with 90% credibility intervals (CrIs) for fenbendazole (FBZ), ivermectin (IVM) and moxidectin (MOX) for *Nematodirus* spp. Using the eggCounts (EC) and bayescount (delta method, BC-D) R packages, the 90% CrIs were calculated. While the eggCounts package also provides an estimate for the FECR, the bayescount package only provides the CrI. Therefore, the FECR was calculated simply from mean pre and post egg counts. For sheep, the grey zone is located between 95% and 99% FECR and indicated by vertical lines. Colours indicate susceptible (green) and resistant (red) results. (For interpretation of the references to colour in this figure legend, the reader is referred to the Web version of this article.)

Faecal egg counts were obtained using the Mini-FLOTAC method with a multiplication factor of 5 (11 farms) or 2.5 (1 farm) for both, pre- and post-treatment samples. The multiplication of 2.5 was necessary on one farm since the raw egg count threshold of 200 was not achieved for the pre-treatment samples after a single round of Mini-FLOTAC analysis and all samples were analysed a second time (four counting chambers per sample). The FECR was calculated using eggCounts for paired samples assuming a common FECR for all samples on a farm without zero-inflation in eggCounts (Wang et al., 2018). The 90% and 95% CrIs were obtained from the quantiles of the posterior distributions. In addition, the data were analysed using bayescount (Peña-Espinoza et al., 2016; Denwood et al., 2023) and 90% CrIs were obtained for most samples. The bayescount package allows to first identify the optimal method to discriminate susceptible and resistant parasite communities but results are shown for multiple methods. The 90% CrIs, however, are not calculated for all methods. For the different variants of the Beta Negative Binomial (BNB) methods (Denwood et al., 2019, 2023), only p-values are provided. Thus, all 90% CrIs provided for bayescount results are based on the delta method (Levecke et al., 2018), although this is explicitly marked by the program as a suboptimal method. The optimal method chosen by bayescount was used to classify a parasite community (farm) as susceptible or resistant. In Fig. 1, the FECR with 90% CrIs are shown for all treatments and all 12 farms for strongyle parasites as calculated using eggCounts and bayescount approaches. On farm 4, more than 200 eggs were counted for *Nematodirus* spp. and thus the FECR for this genus was calculated separately for all drugs and is presented in Fig. 2. No attempt was made to identify the *Nematodirus* species based on egg morphology since the fact that enough eggs were detected for FECR analysis was only recognized retrospectively.

**Fig. 3:**
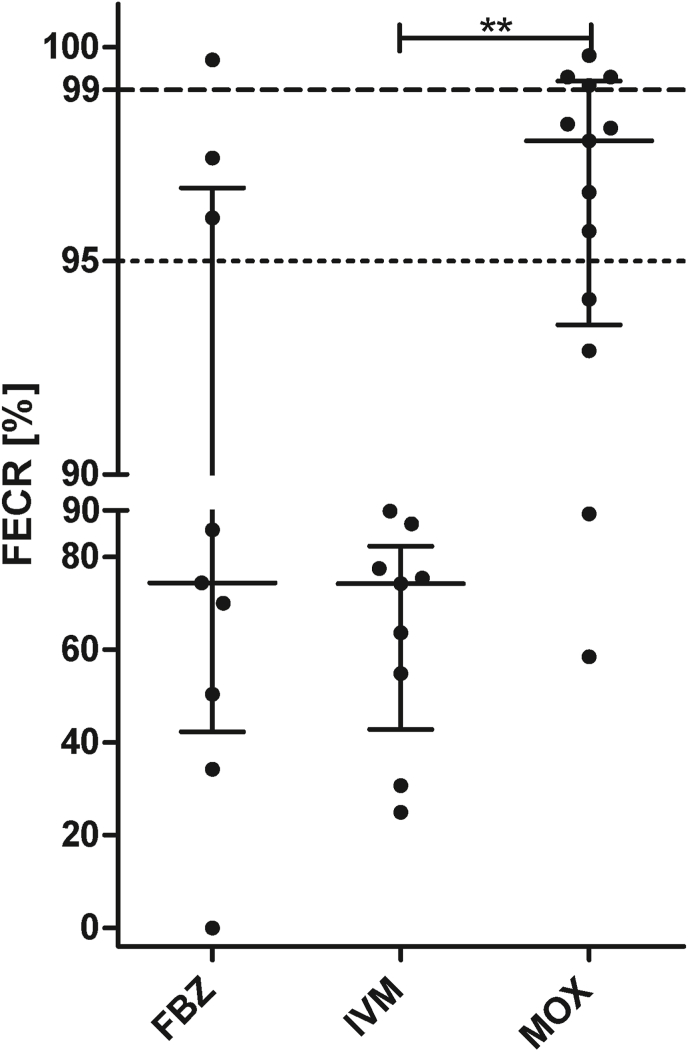
Comparison of strongyle faecal egg count reduction (FECR) values calculated using eggCounts. Each dot represents a different farm/treatment combination. Median and inter-quartile ranges are indicated for treatments with fenbendazole (FBZ) ivermectin (IVM) and moxidectin (MOX). FECR values between different treatment groups were compared using the Kruskal-Wallis test followed by Dunn's post hoc test to compare all groups. **, p < 0.01.

For 87.5% of all results (28/32 comparisons, including one for a treatment with monepantel not included in Fig. 1, Fig. 2), the 90% CrI for the eggCounts method was smaller than the CrI for the bayescount delta method (Fig. S1). In three cases, the bayescount methods produced slightly smaller CrIs and in one case both CrIs had the same width. The difference in CrI width was highly significant (p < 0.0001, Wilcoxon matched-pairs signed rank test) (Fig. S1). Despite this clear difference in the width of the 90% CrIs, the interpretation of the results of the FECRT with both methods was very similar. When farms were categorised as susceptible, inconclusive and resistant (including low resistant), 22 and seven results were assigned by both methods to the resistant and susceptible groups, respectively. One result was considered to represent an inconclusive data set by bayescount and a susceptible one by eggCounts. In addition, two samples were assigned to the susceptible status by bayescount but resistant by eggCounts. Using these classifications, a Cohen's κ value was calculated. The value of 0.774 corresponds to a substantial agreement. Remarkably, there was a complete inter-rater agreement for FBZ and IVM, where most populations showed clear resistance. In contrast, the resistance status for MOX was typically less pronounced, i.e. FECR values were significantly higher than for IVM (Fig. 3). Although the median FECR for FBZ was as low as for IVM, this difference was not significant to MOX since FECR results were highly variable with FECR ranging from 0% to almost 100% (Fig. 3). Therefore, it was expectable that there was more disagreement between both raters since FECR estimates and 90% CrIs were located close to the thresholds for resistance.

Interpretation of the results for FBZ was identical on all eight farms (eight datasets for strongyle and one for *Nematodirus* type eggs) for eggCounts and bayescount. Table 1, Fig. 1, Fig. 2 show details regarding the results. On seven of these farms, strongyle communities were clearly resistant. Only on farm 6, the result of the FECRT indicated a susceptible strongyle population independent of the statistical evaluation method applied (Fig. 1, Table 1). On farm 4, *Nematodirus* spp. were also found to be susceptible for FBZ (Fig. 2, Table 1).

**Table 1:** Comparison of interpretation of the faecal egg count reduction results according to the WAAVP guideline 1992 (only eggCounts statistics) and 2023 (eggCounts vs bayescount statistics).

				Interpretation of FECRT results			
Farm	Parasites	Drug	N^a^	eggCounts/original guideline	eggCounts/revised guideline	Bayescount/revised guideline	MDR^b^
1	Strongyles	MOX	23	Normal	Low resistant	Susceptible	n.a.
2	Strongyles	FBZ	16	Reduced	Resistant	Resistant	Yes
	Strongyles	IVM	17	Reduced	Resistant	Resistant	
	Strongyles	MOX	19	Reduced	Resistant	Resistant	
	Strongyles	MON	20	Normal	Susceptible	Susceptible	
3	Strongyles	FBZ	15	Reduced	Resistant	Resistant	Yes
	Strongyles	IVM	16	Suspected	Resistant	Resistant	
	Strongyles	MOX	15	Suspected	Resistant	Resistant	
4	Strongyles	FBZ	20	Reduced	Resistant	Resistant	Yes
	Strongyles	IVM	20	Reduced	Resistant	Resistant	
	Strongyles	MOX	20	Suspected	Resistant	Resistant	
	*Nematodirus*	FBZ	20	Normal	Susceptible	Susceptible	
	*Nematodirus*	IVM	20	Reduced	Resistant	Resistant	
	*Nematodirus*	MOX	20	Normal	Susceptible	Susceptible	
5	Strongyles	FBZ	38	Reduced	Resistant	Resistant	Yes
	Strongyles	IVM	40	Reduced	Resistant	Resistant	
	Strongyles	MOX	39	Suspected	Resistant	Resistant	
6	Strongyles	FBZ	20	Normal	Susceptible	Susceptible	No
	Strongyles	IVM	20	Normal	Resistant	Resistant	
	Strongyles	MOX	10	Normal	Susceptible	Susceptible	
7	Strongyles	FBZ	22	Reduced	Resistant	Resistant	Yes
	Strongyles	IVM	19	Reduced	Resistant	Resistant	
	Strongyles	MOX	20	Reduced	Low resistant	Low resistant	
8	Strongyles	MOX	17	Normal	Resistant	Resistant	n.a.
9	Strongyles	FBZ	22	Reduced	Resistant	Resistant	Yes
	Strongyles	IVM	19	Reduced	Resistant	Resistant	
	Strongyles	MOX	24	Suspected	Susceptible	Inconclusive	
10	Strongyles	FBZ	20	Suspected	Resistant	Resistant	Yes
	Strongyles	IVM	23	Reduced	Resistant	Resistant	
	Strongyles	MOX	23	Normal	Low resistant	Susceptible	
11	Strongyles	MOX	30	Normal	Susceptible	Susceptible	n.a.
12	Strongyles	MOX	26	Normal	Susceptible	Susceptible	n.a.
					Cohen's κ eggCounts vs. bayescount	0.774 (substantial)	
					Cohen's κ Guidelines 1992 vs. 2023	0.444 (moderate)	

FBZ, fenbendazole; IVM, ivermectin; MOX, moxidectin; MON, monepantel; MDR, multi-drug resistance.

a Number of animals in each treatment group.

b Multi-drug-resistance, defined as resistance to two or more drugs from at least two different drug classes.

Ivermectin efficacy was also evaluated on eight out of 12 farms. Resistance was observed on all eight farms using both statistical methods. In addition, the *Nematodirus* spp. community on farm 4 was also found to be IVM resistant with both statistical methods.

Efficacy of MOX was tested on all twelve farms included in the present study. In general, the FECR values for MOX were higher than for the other drugs and on most farms still above 90% but on farm 2 the FECR was below 60%. Nevertheless, only 3/12 and the *Nematodirus* spp. on farm 4 were susceptible to MOX according to both statistical analysis methods (Table 1). On five farms the strongyles were found to be resistant with eggCounts and bayescount. On farm 1, the strongyles were judged to be resistant with eggCounts and susceptible with bayescount. On farm 9, the interpretation of the FECR was inconclusive using bayescount while eggCounts concluded that the strongyles were susceptible.

Two farmers with high levels of MOX resistance on their farm, an additional FECR with MON were offered. However, only one of the farmers (farm 2) agreed to participate. On this farm, MON was still fully active (Table 1).

#### 3.2.2 Frequency and extent of anthelmintic resistance and occurrence of multi-drug resistance

For the following comparisons, only the results of the eggCounts package were used. Regarding frequency of anthelmintic resistance, 100% (95% CrI 70.1%–100%) of the tested farms showed resistance of the strongyle or *Nematodirus* spp. community to IVM and 77.7% (95% CrI 45.3–93.7%) showed resistance to FBZ. Even for MOX, 69.2% (95% CrI 42.4–87.3%) of the farms were considered to have resistant worm populations. Pairwise comparisons of frequency of farms with resistant strongyle and *Nematodirus* communities using the mid-p exact test showed no significant differences in the frequency of resistant communities.

However, when looking at the extent of anthelmintic resistance in terms of the actually observed FECR value, there were significant differences between the drugs (Fig. 3). While median values for FBZ (74.4%) and IVM (74.6%) were very similar, the distribution was considerably different between both drugs with a much broader inter-quartile range for FBZ (Fig. 3). While all FECR values for IVM were between 24.6% and 89.9%, the range for FBZ was from 0% to 99.95% with two data sets showing reductions above 99% and another three above 95%. For MOX, the median FECR was 97.8% (range 58.2%–99.8%) and this was significantly higher than for both other drugs (Fig. 3).

For the purpose of the study, multi-drug resistance (MDR) was defined as presence of resistance against at least two drugs from two different drug classes. On 6/8 farms on which three (or four) drugs were tested, there was MDR of strongyles, i.e. resistance on 5/8 farms against FBZ, IVM and MOX and on 1/8 farms resistance against IVM and FBZ (Table 1). On farm 6 and for the *Nematodirus* spp. on farm 4 only IVM resistance was observed. Remarkably, none of the eight farms showed susceptibility to all three drugs (Table 1).

#### 3.2.3 Comparison of interpretations according to the WAAVP guidelines from 1992 to 2023

Finally, the interpretation of the FECRT using the previous WAAVP guideline for the detection of anthelmintic resistance (Coles et al., 1992) was compared with the recently published revised guideline (Kaplan et al., 2023) (Table 1). For this purpose, the categories for the detected anthelmintic efficacy from the previous guideline, i.e. reduced, suspected, and normal, were considered to be equivalent to resistant, inconclusive and susceptible from the current guideline (see Fig. 1 in Denwood et al., 2023). For all the 32 comparisons in Table 1, 22 (68.8%) resulted in identical interpretation using the criteria from both guidelines. Of these 22 results, 15 identified parasite communities as resistant and 7 as susceptible. For ten datasets, interpretation of the same eggCounts calculations was different: There were nine communities considered to be resistant according to Kaplan et al. (2023) of which four and five were judged to be normal and suspected according to (Coles et al., 1992), respectively. The inter-rater agreement between both interpretation schemes measured as Cohen's κ coefficient was only moderate (Cohen's κ = 0.444).

### 3.3 Drug use on other relevant management parameters on the farms according to questionnaire data

Data are provided in more detail in Table S3. On 10/12 farms, sheep were the only ruminants, while on farm 3 cattle and goats and on farm 12 goats were also kept. Also, on 10/12 farms, deworming was performed by the farmer, on one farm sometimes by the farmer and sometimes by the veterinarian (farm 11) and on farm 9 the farmer was a veterinarian. On all farms, deworming was planned by the farmer but nine of them dewormed only after advice by a veterinarian or the sheep health service. On seven of the farms the animals were moved to a fresh pasture after deworming. All except of one farm dewormed the animals regularly and only the farmer on farm 5 stated to deworm only when required. This farmer stated in the questionnaire that he made treatment decisions based on examination of the faeces and his visual assessment of the condition of the animals. Since the farmer also stated that his animals were not diagnosed for parasitic nematodes based on coproscopy, he presumably meant presence of diarrhoea and apparent health status of the animals. Faecal examinations were at least sometimes performed on 4/12 farms but only three farmers considered them to be useful. Only on farm 1 and farm 10 a scale was used to determine the weight of the animals before deworming while on all other farms the weight was estimated by visual inspection. The number of dewormings per year ranged for lambs from 2 to 5 times and for older animals between 0 and 5 times. Moxidectin was used on 11 farms, IVM on 7 and doramectin on two farms, only on farm 3 no ML was used. The BZs were used on four farms in total, three used FBZ and three albendazole. On farm 5 and farm 12, levamisole and MON were also applied. No statistical analyses were performed since resistance was present on the majority of farms and for most management practices were very similar between farms. For some of the variables such as deworming frequency there was no obvious correlation with the resistance status.*3.4. Strongyle nematode species composition*.

To calculate the nemabiome composition of the hatched L1, deep amplicon sequencing of the ITS-2 region was performed. Between 33,644 and 117,934 paired end reads were generated. After trimming, filtering, read merging and removal of chimeras, the number of reads obtained per sample was between 13,520 and 48,231 (median 18650) (Table S4). The numbers of filtered reads were multiplied with the species-specific correction factors to obtain abundance data. From the latter, the frequencies of the parasites in the different samples were calculated.

#### 3.3.1 Pre-treatment composition of strongyle communities

**Fig. 4:**
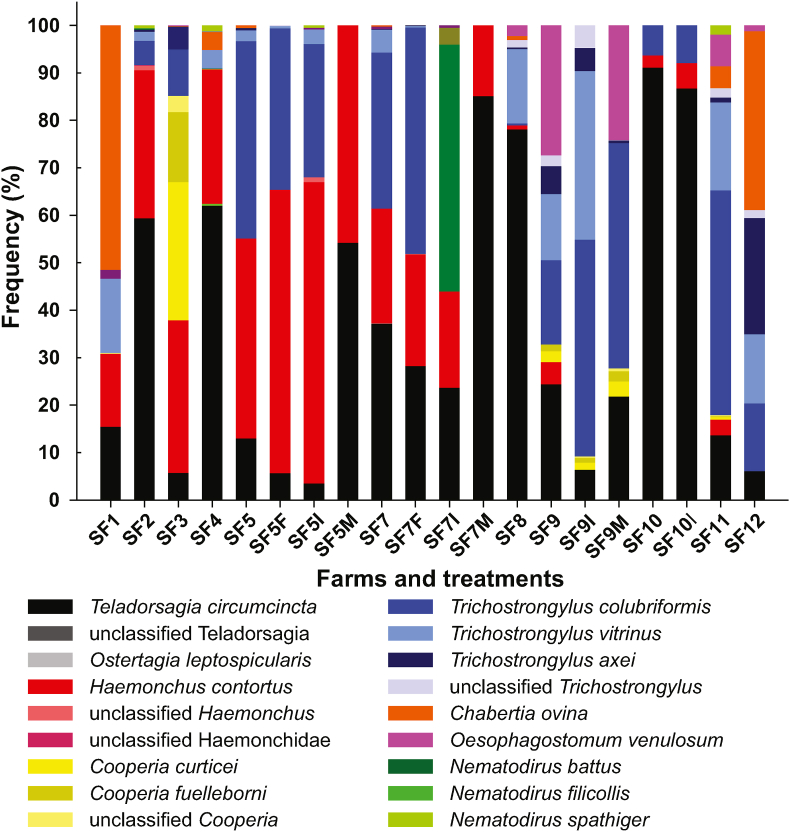
Species composition on farms using deep amplicon sequencing data. The reads were multiplied with species-specific correction factors and these data were used to calculate frequencies for all samples. The samples are labelled indicating the sheep farm (SF1, SF2, SF3, etc.) and the drug used for treatment (F, fenbendazole; I, ivermectin; M, moxidectin). Pre-treatment samples have no additional label except of the farm identity.

Pre-treatment samples for deep amplicon sequencing were obtained from eleven farms but were missing for farm 6 since egg isolation failed. In total, nine post-treatment samples could be analysed, which originated from four different farms. Post-treatment samples in general had lower epg values and thus purification of eggs was not always successful even though FECR results <99% indicated resistance. The composition of strongyle parasite communities in pre and post-treatment samples is shown in Fig. 4. The number of observed species in samples before treatment ranged between 4 (farm 10) and 12 (farm 11) (Fig. S2). This is based on read numbers corrected according to (Redman et al., 2019) and includes four not completely identified classifications such as “unclassified *Haemonchus*” or “unclassified Haemonchidae”, which had a mean frequency of 0.1% in all samples (range 0–1.0% in the different samples). There were also reads included corresponding to three *Nematodirus* species with a mean frequency in all samples of 0.4% (range 0–1.9%), which is remarkable since *Nematodirus* larvae should not hatch under these experimental conditions and therefore be absent from the samples. The iNEST function from the iNEXT package 3.0.0 applying a Hill number of zero and two was used to estimate species richness (Chao1) but for all samples the estimated number of species in the samples using rarefaction was exactly the number of observed sequences with all 95% CIs <0.001. This suggests that additional sequencing depth is unlikely to detect additional species in the samples.

*Teladorsagia circumcincta* was the only nematode species that occurred on all eleven investigated farms before treatment (Fig. 4) with a mean frequency of 36.9% (Table S5). It was also the most frequently observed parasite on six of the farms with frequencies >50% on four of them. The second most frequently found parasite was *T. colubriformis*, which was also detected on 10/11 farms but was missing on farm 1. The mean frequency on the farms was 16.0%. It was the most frequently found parasite on one farm. The mean frequency of *H. contortus* was 16.8% and it was found in all pre-treatment samples except for farm 12. It was the most frequent parasite on two farms. On some farms, parasites with an overall rather low frequency were very frequent. *Chabertia ovina* had a pre-treatment frequency of only 9.0% but was the most frequent parasite on two farms. *Oesophagostomum venulosum* and *C. curticei* were the most frequent parasites on one farm each but their mean frequency on all farms was only 3.4% and 2.9%, respectively.

#### 3.3.2 Effects of anthelmintic treatments on species composition

For two farms (SF5 and SF7), data were available for pre-treatment and after treatment with all three drugs. On both farms, frequency of *T. circumcincta* decreased after treatment with FBZ and IVM but increased after treatment with MOX. The frequency of *H. contortus* slightly increased after FBZ, IVM and MOX treatment on farm 5 while on farm 7 it remained the same after FBZ treatment but decreased after IVM and particularly after MOX treatment. For *T. colubriformis*, a decrease in frequency was observed after treatment with FBZ and IVM on SF5 while it increased on SF7. On both farms, *T. colubriformis* completely disappeared after MOX treatment. *Trichostrongylus vitrinus* also survived treatments with FBZ and IVM but was eliminated by MOX treatment. Fenbendazole considerably reduced the frequency of *T. vitrinus* whereas the frequencies were quite stable after treatment with IVM. *Chabertia ovina* was present at frequencies below 1% before treatment and was eliminated by all three treatments. On SF9, post treatment data for both MLs were available. On this farm, *T. circumcincta* frequencies decreased and slightly decreased after IVM and MOX treatment, respectively. In contrast, there was a strong increase in the frequency of *T. colubriformis*. *Haemonchus contortus* and *T. vitrinus* were completely susceptible to both MLs. Frequency of *T. vitrinus* strongly increased after IVM treatment but the parasite was absent after MOX treatment. In contrast, some *C. curticei* and *C. fuelleborni* survived treatment with both drugs. Remarkably, *O. venulosum* was fully susceptible to IVM but its frequency barely changed after MOX treatment. On farm 10, *T. circumcincta*, *H. contortus* and *T. colubriformis* were the only parasites present before and post treatment and frequencies only slightly changed after IVM treatment.

Species diversity was calculated as inverse Simpson index for all samples as shown in Fig. S2. When groups were defined by drugs (Fig. S2A), there was a clear tendency for lower species diversity in post-treatment samples but the number of samples for the individual drugs was too low for any statistical analysis. Therefore, the three post-treatment groups were pooled. In this setting, there was a significant decrease in species richness using the inverse Simpson index after treatment applying a Wilcoxon matched-pairs signed rank test (Fig. S2B).

**Fig. 5:**
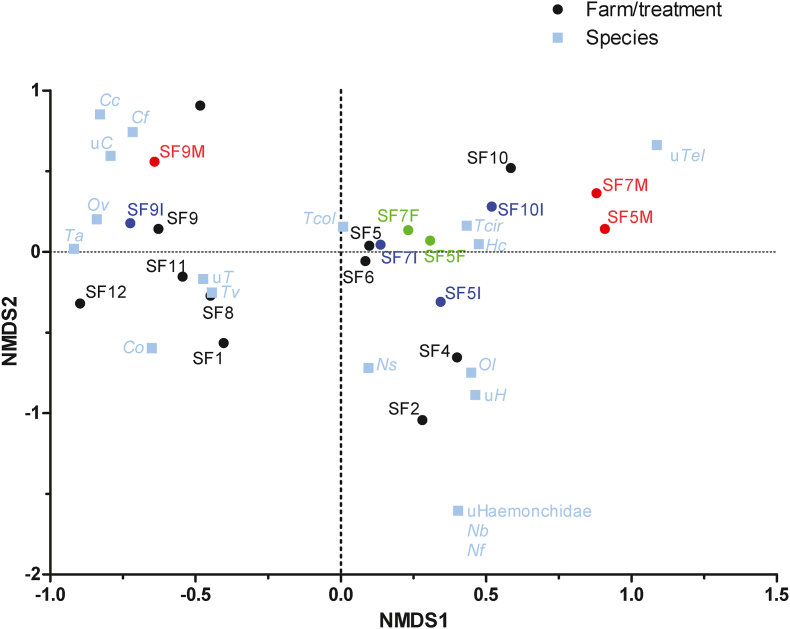
Analysis of species β-diversity using ordination by non-metric multidimensional scaling (NMDS). A dissimilarity matrix was calculated using the Bray-Curtis dissimilarity method. The NMDS ordination plot shows no obvious pattern for any of the treatments. Samples are labelled indicating the farm (SF1, SF2, SF3, etc) and the drug used for treatment (F, fenbendazole (green); I, ivermectin (blue); M, moxidectin (red)). Pre-treatment samples are shown in black. (For interpretation of the references to colour in this figure legend, the reader is referred to the Web version of this article.)

In order to identify potential general patterns of treatments with different drugs, an NMDS analysis was performed. For this purpose, frequency data were used. The NMDS plot shown in Fig. 5 does not reveal any specific effects of treatment. The post-treatment samples were not located close together in the ordination plot (Fig. 5). The first dimension of the plot (x axis) is dominated by the location of *H. contortus* and *T. circumcinta* on the right and *T. axei*, *O*. *venulosum* and also *T. vitrinus* on the left site while *T. colubriformis* is very close to the centre of the plot. The second dimension (y axis) is dominated by less abundant species, i.e. unclassified Haemonchidae and *Nematodirus* spp. on the bottom, *C. ovina* (lower left) and *Cooperia* spp. (upper left) and unclassified *Teladorsagia* (upper right). Many of the samples post treatment with IVM or FBZ are located close to the triangle with *H. contortus*, *T. circumcincta* and *T. colubriformis* at the corners reflecting the fact that these three species frequently survived these treatments while other species were typically missing post treatment. SF9 with high frequency of *O. venulosum* before and after MOX treatment and high frequency of *T. axei*, and an increase of *T. colubriformis* after MOX treatment is immediately identifiable and an exception on the NMDS plot. In addition, cluster analysis using hierarchical clusters and k-means clustering did not suggest any convincing patterns in frequency data.

## 4 Discussion

Anthelmintic resistance of sheep strongyle nematodes is known to occur worldwide and has also been reported for multiple central and northern European countries such as the Netherlands, Belgium, Sweden, Denmark and Austria (Rose et al., 2015; Ploeger and Everts, 2018; Claerebout et al., 2020; Beleckė et al., 2021; Untersweg et al., 2021) including Germany (Voigt et al., 2022). However, a recent meta-analysis revealed that there are considerable gaps in our knowledge (Rose Vineer et al., 2020). The finding that resistance to at least one drug on every farm where three different treatments were evaluated is therefore not surprising, but nevertheless alarming. In general, among the three drugs tested in the present study, MOX is expected to be least affected by resistance issues. Although this was indeed observed in terms of significantly higher FECR values and a (not significantly) lower frequency of resistance on the farms, the frequency of farms with resistance and low resistance was almost 70%. Since frequency of resistant parasites can rapidly increase after only a few treatments once resistant parasites are present and an adequate reservoir is missing (Knapp-Lawitzke et al., 2015), it is reasonable to assume that MOX resistance will rapidly increase in the future. Farmers will be tempted to use it more frequently than products with e.g. FBZ or IVM since it still leads to clinical improvement of the animals, which is likely not the case for treatments with BZs and IVM on farms with low FECR concerning these compounds.

Several approaches are available to calculate CIs/CrIs from FECRT data. The classical approach described by Coles et al. (1992) should not be used nowadays since it neither takes the paired data structure (before and after treatment as recommended in the recent guideline by Kaplan et al. (2023) into account nor are the different sources of variation (variation between replicate counting chambers, within a faecal sample, within an animal over the day and between animals in the group) modelled appropriately. There are two R packages available that have been written to estimate these variances and provide more realistic CIs/CrIs, i.e. eggCounts and bayescount. Both packages rely on Bayesian models to estimate credible limits from the data. Here the outcomes for both packages were compared regarding the width of the 90% CrIs and the assignment of a sample to a certain resistance status. The 90% CrIs were significantly wider for bayescount compared to eggCounts. Whether this means that bayescount overestimates or eggCounts underestimates the uncertainty is not within the scope of this study and will require comparison based on simulated data. However, when both approaches lead to the same susceptibility/resistance status for a real field sample, one can probably be more confident that this assignment is correct. Remarkably, this was the case for 29/32 comparisons between pre- and post-treatment data sets in the present study. The inter-rater agreement calculated as Cohen's κ also indicated substantial agreement between both methods. All cases in which both methods disagreed involved treatments with MOX, which in general resulted in higher FECR values than IVM and FBZ treatments and where assignments to the subcategory low resistance occurred. This high degree of agreement suggests that other new recommendations included in the current guideline (Kaplan et al., 2023), in particular the fact that at least 200 eggs should be counted under the microscope for pre-treatment samples, led to robust data that allowed reliable estimates of the FECRs.

It was also aimed to compare what effects the interpretation rules in the previous (Coles et al., 1992) and current guideline (Kaplan et al., 2023) on the assignment of a resistance status would have. Here it must be emphasised that the original guideline was expected to be used with unpaired data, i.e. comparison between a control and a treatment group on the same day post treatment, while the revised guideline strongly recommends to use paired data comparing data from the same animals before and after treatment. However, the cut-offs postulated by (Coles et al., 1992) were already applied to paired samples for a long time and thus the concept of using these cut-offs on paired data is not at all new for the present study. Even before the publication of the original guideline, the potential advantages of a paired study design were discussed (McKenna, 1990). Several methods to calculate the FECR for either treatment and control group data or for pre and post treatment data were evaluated and often paired data analysis was considered to be advantageous and newly developed software package typically included analysis options for paired data (Schnyder et al., 2005; Torgerson et al., 2005, 2014; McKenna, 2006; Denwood et al., 2010; Dobson et al., 2012). In all these cases, the criteria proposed by Coles et al. (1992) based on the estimate of the FECR and the lower 95% CL were applied. Therefore, the paired data analysis is not really the new aspect in the concept of the revised guideline. Indeed, the new concept of the revised guideline is based on the switch to use only the upper and lower 90% credible limits and not the lower 95% confidence limit and the estimate of the FECR as criteria to assign a susceptibility/resistance status. The second really new idea of the revised guideline is the reliance of the interpretation on an expected efficacy (efficacy of drug in the studies conducted for licensing purposes) as the upper limit and a lower efficacy limit specified by an, actually arbitrarily chosen, non-inferiority margin. This means the level of decreased efficacy observed that we are willing to accept to be not appreciably worse than the expected efficacy (Denwood et al., 2023).

For sheep the lower and upper thresholds used to assign FECRT values to a resistance status were raised from 90 to 95%–95% to 99% (Denwood et al., 2023; Kaplan et al., 2023). Agreement of assignments using criteria from the original and revised guideline was observed for 22/32 comparisons of pre- and post-treatment data and the Cohen's κ value suggests only moderate agreement. Thus, the change in the criteria to assign data to a certain resistance status had a higher effect on the interpretation than the use of two different statistical methods, eggCounts and bayescount. This only moderate agreement between interpretation of data by the previous and current guideline has considerable effects on comparison of results from new and older studies that applied different criteria to evaluate FECR data. In principle, it should be recommended to also provide the estimate of the FECR value and its 95% CrIs. Without providing such data, it will be impossible to compare recent data with descriptions of the resistance status from a few years ago. An obvious effect of applying the criteria of the current guideline is that the frequency of detection of resistant gastrointestinal nematodes will considerably increase. The higher sensitivity of the revised study design obviously leads to less inconclusive results and allows earlier detection of emerging resistance.

In terms of species diversity, there was an overall significant decrease in diversity, measured as inverse Shannon index, by treatment. This effect was not statistically significant for individual treatment groups since the number of post-treatment samples for individual drugs was only in the range of two to four. Despite this overall effect on diversity, the NMDS approach did not reveal any obvious similarities of post-treatment groups. This can be simply explained by two facts: First, treatments with FBZ and IVM were very inefficient and not unexpectedly resistance of the strongyle community was not limited to one or two species. Secondly, the effects of the more effective MOX were highly divergent on different farms. On SF5 and SF7, only *H. contortus* and *T. circumcincta* (plus of a few unclassified *Telardorsagia* spp. on SF7) survived MOX treatment while *T. colubriformis* was fully susceptible. In contrast, *H. contortus* was fully susceptible on SF9 whereas *T. circumcincta* but also *T. colubriformis*, *O*. *venulosum*, *C. curticei* and *C. fulleborni* apparently showed high levels of MOX resistance. These very different responses in addition to very different pre-treatment strongyle communities explain why no similar β-diversity patterns were observed after treatment.

On four of the farms, *Nematodirus* spp. were identified in the metagenome data. This is unexpected since L1 of this genus do not hatch from the eggs. However, the method used to prepare L1 and their DNA does not exclude that DNA from *Nematodirus* spp. is co-purified with DNA from strongyle L1. There was no additional step to remove unhatched eggs from L1 since such a step, e.g. using a Baermann funnel, would have reduced the number of collected larvae further without completely excluding unhatched eggs.

It should in general be kept in mind that deep-amplicon read counts do not necessarily reflect worm count data and there are multiple confounders such as: (i) different fecundity of female worms between species: (ii) different ability of L1 to further develop to L2 if small amounts of bacteria are still present in the purified egg suspension, (iii) unequal PCR efficacy for different nematode species, (iv) different copy numbers of the ITS-2 region in the genome, (v) different numbers of cells in the investigated parasite stages (L1 here and in the original nemabiome protocol). The experimentally determined correction factors aim to address the confounders listed under iii-v. It will not be possible to correct for differences in fecundity since this might be affected by the host's immune status, parasite density, other parasite species in the same or even other compartments of the host, the age of the parasites, i.e. fecundity of females may vary over time, and the ratio of female to male worms. Also, the chances that some of the L1 will develop into L2 cannot be modelled and will depend on various factors including the skill of the person conducting the experiment to obtain highly purified eggs, the faecal consistence (e.g. sheep vs. cattle, animal with and without diarrhoea), the epg, the host species and the parasite species. Even after correction using experimentally determined correction factors, the corrected abundance or frequency data do not represent true frequencies of the larvae of different parasite species. The correction factors are only available for six important sheep parasites (*H. contortus*, *T. axei*, *T. colubriformis*, *T. vitrinus*, *T. circumcincta* and *C. curticei*, ordered from low to high correction factor) and ranged from 0.70 to 1.61) (Redman et al., 2019). For all other species, no correction factors are available at present and thus, the actually used correction factor is 1.0. In the current data set (including pre- and post-treatment data), the proportion of reads that were assigned to taxa other than the six with available correction factor was 28.7%. The fact that no correction factors are available for these species clearly shows that abundance data calculated from read frequencies suffer from uncertainties particularly if parasites other than those for which correction factors were established are among the more abundant species. In the present data set this is particularly the case for SF1 (>50% corrected abundance of *C. ovina*), SF3 (14.7% *C*. *fuelleborni* before treatment), SF9 (about 27% *O. venulosum* before treatment and about 24% post-treatment with MOX).

Despite all these limitations, nemabiome data nevertheless represent the best option we currently have to characterise strongyle nematode communities. It is the only method that allows to investigate strongyle nematode communities from faecal on a species level without making assumptions about their composition and the shear amount of data that can be produced very easily allows to detect also very rare species that so far are barely investigated. It also at least to some extend provides quantitative data. Its limitations regarding for instance variation of copy number of target genes do also apply to most other meta-barcoding methods where they are typically ignored. Making the effort to establish correction factors is already a considerable improvement in comparison to many studies using environmental DNA to characterise species communities in ecosystems. In most of these studies, read counts need to be used directly as proxy for abundance.

Despite a relatively limited geographical range in which the farms were localized, and a very limited number of post-treatment samples for which nemabiome data could be obtained, completely different populations of drug resistant strongyle species were observed on the farms. This would be in accordance with resistance arising locally on different farms and involving multiple species occurring on these farms. Based on the questionnaire survey conducted herein, on most of the farms during routine anthelmintic treatments animals were not weighed on a scale but their weight was estimated to determine the drug dosage. This suggests that actual dosages were varying widely and that a lot of the animals were underdosed, which would facilitate the evolution of drug resistance. Other practises on farms that might promote local evolution of drug resistance include dose-and-move strategies (7/12 farms), treating all animals on the farm simultaneously (9/12 farms), both minimizing the refugium of susceptible nematodes, and high treatment frequencies (up to every five weeks) and lack of coproscopical examinations, which leads to exposure of parasites to drugs in clinically healthy hosts.

As an alternative to local evolution of resistant parasite populations on the farms, the overall distribution of drug resistance of different species could also be easily explained by high levels of animal movement between farms without considering any effective quarantine measures. Although 10/12 farmers stated that they separated newly bought animals, only 7/12 also treated the new animals with an anthelmintic. If none of these measures was combined with coproscopic diagnosis to detect nematode infections, they are probably only poorly effective to prevent introduction of parasites with anthelmintic resistance into farms. Under such conditions, resistance of individual parasite species might arise on different farms and then spread rapidly by barely controlled movement of animals between farms. In order to distinguish between both possible explanations, a much higher number of included farms, a random sampling strategy based on geographic (grid-based) structuring and presumably also more reliable data about movements of animals between farms would be required. For this purpose, more financial resources, a much higher willingness of farmers to participate in sampling and sharing of objective data on management practices (treatments, quarantines, etc.) as well as detailed information about animal movements would be required. There would still be confounding factors such as spread of resistant parasites using other susceptible livestock (cattle, goats, camelids) or wild cervids (roe deer) (Brown et al., 2022). However, studies applying geographical/spatial analysis methods would definitively improve our understanding of the processes underlying spread of resistant populations in small ruminants.

On farm 9 there was an unexpected effect of the anthelmintic treatments. On this farm, post-treatment species data are available for IVM and MOX. The strongyle community on this farm was considered to be IVM resistant (90% CrI FECR 87.4–92.0% with eggCounts) while it was considered MOX susceptible (90% CrI FECR 94.9–99.9%). For IVM, the interpretation was confirmed by bayescount while the MOX interpretation with bayescount was inconclusive. On this farm, *T. vitrinus* was resistant to IVM but fully susceptible to MOX. In contrast, *O. venulosum* was completely eliminated from the sheep by IVM treatment but its frequency remained almost constant after MOX treatment. In general, it is assumed that IVM resistance evolves before MOX resistance. Here, it is possible that the opposite happened. However, one must be very careful when interpreting these data. First of all, there was no proven MOX resistance on the farm and it should therefore not be stated that the parasites were resistant. Due to the high FECR after MOX treatment, there were only very few surviving larvae and this might in addition lead to stochastic drift. Even without such random effects, the high frequency of *O. venulosum* does not necessarily indicate that the FECR for *O. venulosum* was below 99%. The upper 90% credible limit was 99.9%. With 24% of the surviving L1 identified as *O. venulosum* and barely no change caused by treatment, the FECR for this species might still be above 99%. It would be necessary to establish a single-species isolate and characterise this in vivo and ideally also in vitro in comparison to susceptible field isolates. It was aimed to obtain such a field isolate but this failed so far. Unfortunately, the farmer on farm 9 has in the meantime given up sheep breeding due to financial issues and it will no longer be possible to establish an *O. venulosum* isolate from this parasite community.

Although the efficacies of IVM and FBZ were already poor on most farms, efficacies for FBZ were even slightly worse than those for IVM. Previous studies on the occurrence of anthelmintic resistance in sheep in Germany are scarce and all had certain limitations. In 2001/2002, a study showed resistance against FBZ, defined as mean FECR <95% on 19/28 (66.6%) farms in Lower Saxony (Moritz, 2005). In a study in seven German federal states, 9/53 (16.9%) showed evidence of MOX resistance (Perbix, 2008) applying the criteria proposed in the original WAAVP guideline (Coles et al., 1992). Scheuerle et al. (2009) reported resistance against MOX, albendazole and oxfendazole on a farm in Bavaria, while for a farm in Baden-Wuerttemberg resistance against albendazole and FBZ but full efficacy of MOX was observed. Based on pooled faecal samples chosen and applied by the farmer or the local veterinarian, Voigt et al. (2022) classified treatments as efficient if the FECR was ≥95%. They reported lower efficacy for 23/44 (52.3%) farms for BZs, 3/5 (60.0%) farms for avermectins, 39/87 (45.3%) farms for MOX. Moreover, the authors reported low efficacies of MON, levamisole and a closantel/mebendazole combination on 10–15% of the farms (Voigt et al., 2022). In many of these studies the weight of the sheep was only estimated or the studies do not provide a description how the weight was determined. Only two of them applied the (original) WAAVP guideline (Perbix, 2008; Scheuerle et al., 2009). Nevertheless, these data at least suggest that resistance to all anthelmintic drug classes is increasing in Germany over the years.

In the present study, one to three different MLs were used on all the farms whereas BZs were used on only four farms (33%). This can probably be explained by a switch of farmers from BZs to MLs when the first problems with BZ resistance occurred. Since the introduction of the MLs in the market, the only new sheep anthelmintic class that has been introduced in the German market are the aminoacetonitrile derivatives with its only commercialised representative monepantel (Kaminsky et al., 2008; Sager et al., 2009b). This was used by only 2/12 farmers suggesting that it has so far not achieved a broad market share in Germany. The simple explanation might be its higher price (about 33% higher net purchase price per dose for the veterinarian when compared to the second most expensive drug MOX (source: vetidata.de; last accessed December 27, 2023). Moreover, the product information leaflet explicitly discourages the use of MON more than twice per year. Another drug that is apparently not often used by sheep farmers in Germany is LEV. Due to its small safety margin and its lack of effects against other parasites, most farmers do not regularly use this drug. Both MON and LEV act by opening or facilitation of opening of different nicotinic acetylcholine receptors and resistance to both drugs can be achieved by loss-of-function in the receptor channel subunits (Rufener et al., 2010; Kotze et al., 2014). In particular the latter phenomenon suggests that both drugs will not contribute a long-term solution to the problem of multi-drug resistance if used in a non-sustainable way. For instance, (Raza et al., 2016) were able to select a highly MON resistant isolate from a single field treatment with an efficacy >99% by collecting the few larvae surviving treatment.

In conclusion, the combination of sensitive methods to determine FECs with state-of-the-art statistical methods to estimate variance allowed to determine the extent of anthelmintic resistance and estimate how urgent the emerging problem of MOX resistance is. Further studies also involving efficacy of LEV, MON and closantel will be required to obtain an overlook on the whole dimension of the anthelmintic resistance problem in small ruminants in Germany. Obviously, the sheep breeding industry in Germany is rapidly running into a situation with widespread multi-drug anthelmintic resistance similar to what has been described in several countries in the southern hemisphere such as New Zealand, Australia, South Africa as well as the UK (Gilleard et al., 2021). The nemabiome data show that anthelmintic resistance is obviously not only a problem in the major sheep parasites *H. contortus*, *T. circumcincta* and *T. colubriformis* but might also include other nematode species. Whether this is a problem of only a few farms or more widespread will require more in-depth investigations for which the methodological approaches have now been established.

## Funding

The authors gratefully ackknowledge funding of this project by the Federal Office of Consumer Protection and Food Safety (Contract number: 2019000390)

## CRediT authorship contribution statement

**Jürgen Krücken:** Conceptualization, Data curation, Formal analysis, Funding acquisition, Methodology, Project administration, Supervision, Validation, Visualization, Writing – original draft, Writing – review & editing. **Paula Ehnert:** Data curation, Formal analysis, Investigation, Methodology, Validation, Visualization, Writing – original draft, Writing – review & editing. **Stefan Fiedler:** Data curation, Formal analysis, Investigation, Validation, Visualization, Writing – review & editing. **Fabian Horn:** Data curation, Formal analysis, Validation, Writing – review & editing. **Christina S. Helm:** Conceptualization, Writing – review & editing. **Sabrina Ramünke:** Investigation, Writing – review & editing. **Tanja Bartmann:** Investigation, Writing – review & editing. **Alexandra Kahl:** Investigation, Writing – review & editing. **Ann Neubert:** Investigation, Writing – review & editing. **Wiebke Weiher:** Conceptualization, Funding acquisition, Writing – review & editing. **Ricarda Daher:** Conceptualization, Funding acquisition, Project administration, Writing – review & editing. **Werner Terhalle:** Conceptualization, Writing – review & editing. **Alexandra Klabunde-Negatsch:** Conceptualization, Writing – review & editing. **Stephan Steuber:** Conceptualization, Funding acquisition, Project administration, Writing – review & editing. **Georg von Samson-Himmelstjerna:** Conceptualization, Funding acquisition, Project administration, Supervision, Writing – original draft, Writing – review & editing.

## Declaration of competing interest

The authors declare the following financial interests/personal relationships which may be considered as potential competing interests: Georg von Samson-Himmelstjerna (GvSH) reports financial support was provided by Federal Office of Consumer Protection and Food Safety, Berlin, Germany. GvSH is a member of the editorial board of Int. J. Parasitol. Drugs Drug Rest. Furthermore, he declares that he has previous and ongoing research and consultancy collaborations with several veterinary pharmaceutical and diagnostic companies.
