# Supplementary material for: Faecal egg count reduction tests and nemabiome analysis reveal high frequency of multi-resistant parasites on sheep farms in north-east Germany involving multiple strongyle parasite species
Source: Int J Parasitol Drugs Drug Resist. 2024 May 5;25:100547. doi: 10.1016/j.ijpddr.2024.100547 (PMC11097076; doi:10.1016/j.ijpddr.2024.100547)
Supplement: Supplementary file 1 [file mmc1.pdf]

Table S1

Primer sequences

Primer name	Sequence (5'->3')
NClAdp	TCGTCGGCAGCGTCAGATGTGTATAAGAGACAGACGTCTGGTTCAGGGTTGTT
NClAdplN	TCGTCGGCAGCGTCAGATGTGTATAAGAGACAGNACGTCTGGTTCAGGGTTGTT
NC1Adp2N	TCGTCGGCAGCGTCAGATGTGTATAAGAGACAGNNACGTCTGGTTCAGGGTTGTT
NC1Adp3N	TCGTCGGCAGCGTCAGATGTGTATAAGAGACAGNNNACGTCTGGTTCAGGGTTGTT
NC2Adp	GTCTCGTGGGCTCGGAGATGTGTATAAGAGACAGTTAGTTTCTTTTCCTCCGCT
NC2AdplN	GTCTCGTGGGCTCGGAGATGTGTATAAGAGACAGNTTAGTTTCTTTTCCTCCGCT
NC2Adp2N	GTCTCGTGGGCTCGGAGATGTGTATAAGAGACAGNNTTAGTTTCTTTTCCTCCGCT
NC2Adp3N	GTCTCGTGGGCTCGGAGATGTGTATAAGAGACAGNNNTTAGTTTCTTTTCCTCCGCT
