# Supplementary material for: Faecal egg count reduction tests and nemabiome analysis reveal high frequency of multi-resistant parasites on sheep farms in north-east Germany involving multiple strongyle parasite species
Source: Int J Parasitol Drugs Drug Resist. 2024 May 5;25:100547. doi: 10.1016/j.ijpddr.2024.100547 (PMC11097076; doi:10.1016/j.ijpddr.2024.100547)
Supplement: Supplementary file 2 [file mmc2.pdf]

Table S2

Overview of farms, treatment groups included in the study, animal numbers and sampling time points.

Farm no.	Drug tested	Total number of animals on farm	Number of young animals on farm[a]	Day of post-treatment sampling
1	MOX	48	20	14
2	FBZ	450	120	14
2	IVM			
2	MOX			
2	MON			
3	FBZ	475	175	14
3	IVM			
3	MOX			
4	FBZ	953	517	15
4	IVM			
4	MOX			
5	FBZ	1180	400	13
5	IVM			
5	MOX			
6	FBZ	200	77	14
6	IVM			
6	MOX			
7	FBZ	2000	400	13
7	IVM			
7	MOX			
8	MOX	56	26	14
9	FBZ	160	70	14
9	IVM			
9	MOX			
10	FBZ	3500	1600	13
10	IVM			
10	MOX			
11	MOX	200	30	14
12	MOX	530	30	16

[a]One to two years of age.

MOX, moxidectin; FBZ, fenbendazole; IVM, ivermectin; MON, monepantel.
