# Supplementary material for: Faecal egg count reduction tests and nemabiome analysis reveal high frequency of multi-resistant parasites on sheep farms in north-east Germany involving multiple strongyle parasite species
Source: Int J Parasitol Drugs Drug Resist. 2024 May 5;25:100547. doi: 10.1016/j.ijpddr.2024.100547 (PMC11097076; doi:10.1016/j.ijpddr.2024.100547)
Supplement: Supplementary file 3 [file mmc3.pdf]

Table S3

Farm details and various gastrointestinal-nematode control related management parameters on the tested sheep farms (SF).

Parameter	Farms											
	SF1	SF2	SF3	SF4	SF5	SF6	SF7	SF8	SF9	SF10	SF11	SF12
Other ruminants[a]	N	C, G	N	N	N	N	N	N	N	N	N	G
No. sheep	48	450	475	953	1180	200	2000	56	160	3500	200	530
No. lambs	20	60	75	399	250	47	200	10	35	800	15	30
Always quarantine new animals	Y	Y	Y	Y	Y	Y	Y	N	Y	N	Y	Y
Always deworm new animals	Y	N	N	Y	Y	Y	Y	Y	Y	N	N	N
Deworming strategy[b]	R	R	R	R	I	R	R	R	R	R	R	R
Deworming person[c]	F	F	F	F	F	F	F	F	F=V	F	F, V	F
Deworming planer	F	F	F	F	F	F	F	F	F	F	F	F
Advised planning[d]	N	Y	N	Y	Y	Y	N	Y	N	Y	Y	Y
Dose-and-move[d]	N	Y	Y	Y	N	Y	N	N	Y	Y	Y	N
All sheep at same timepoint[d]	Y	Y	Y	Y	N	Y	Y	N	N	Y	Y	Y
No. dewormings lambs/year	1	2	0	2	0	0	0-1	>6	1	4	2	3-4
No. deworming of older sheep/year	1	0	2	2	0	0	1-2	1	1	0	0	2-5
Faecal examinations[d]	N	N	Y	N	N	Y	N	Y	N	Y	N	N
Faecal examinations helpful[d]	N	Y	Y	Y	Y	Y	N	N	Y	Y	Y	N
Weight determination[e]	S	E	E	E	E	E	E	E	E	S	E	E
Drugs used[d]												
Moxidectin	Y	Y	N	Y	Y	Y	Y	Y	Y	Y	Y	Y
Ivermectin	N	Y	Y	N	Y	N	Y	N	Y	Y	N	Y
Doramectin	N	N	N	Y	N	N	N	N	N	Y	N	N
Fenbendazole	N	N	Y	N	N	Y	Y	N	N	Y	N	N
Albendazole	N	N	Y	N	Y	N	Y	N	N	Y	N	Y
Levamisole	N	N	N	N	Y	N	N	N	N	N	N	Y
Monepantel	N	N	N	N	Y	N	N	N	N	N	N	Y

[a]C, cattle; G, goats; N, none.

[b]R, regularly; I, if required.

[c]F, farmer; V, veterinarian.

[d]Y, yes; N, no.

[e]S, Scale; E, estimate.
