# Supplementary figures and images for: Faecal egg count reduction tests and nemabiome analysis reveal high frequency of multi-resistant parasites on sheep farms in north-east Germany involving multiple strongyle parasite species
Source: Int J Parasitol Drugs Drug Resist. 2024 May 5;25:100547. doi: 10.1016/j.ijpddr.2024.100547 (PMC11097076; doi:10.1016/j.ijpddr.2024.100547)

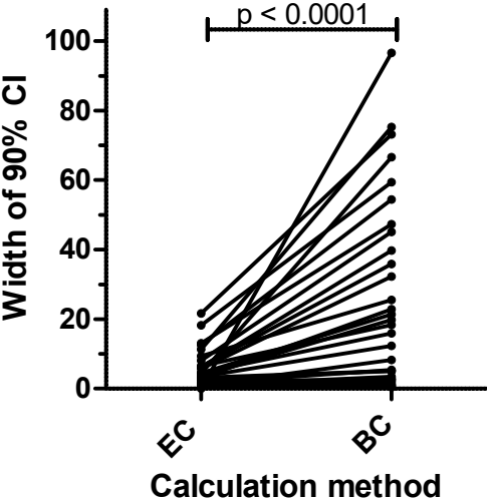

Supplement: Supplementary file 4 [file mmc4.pdf]

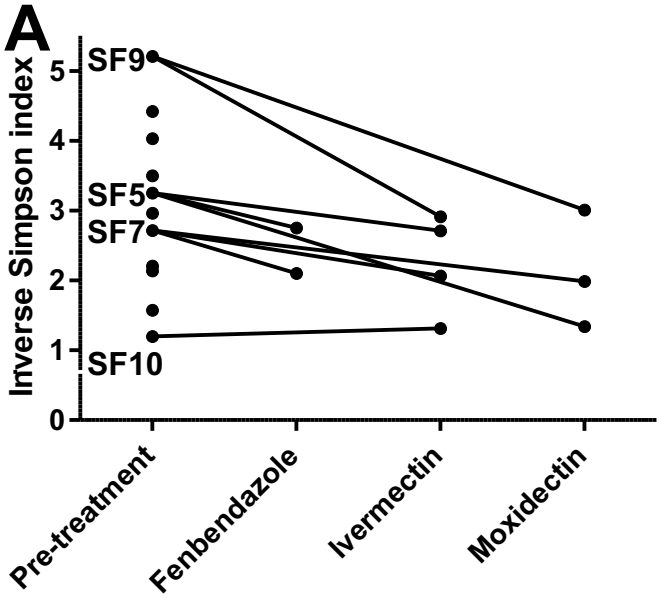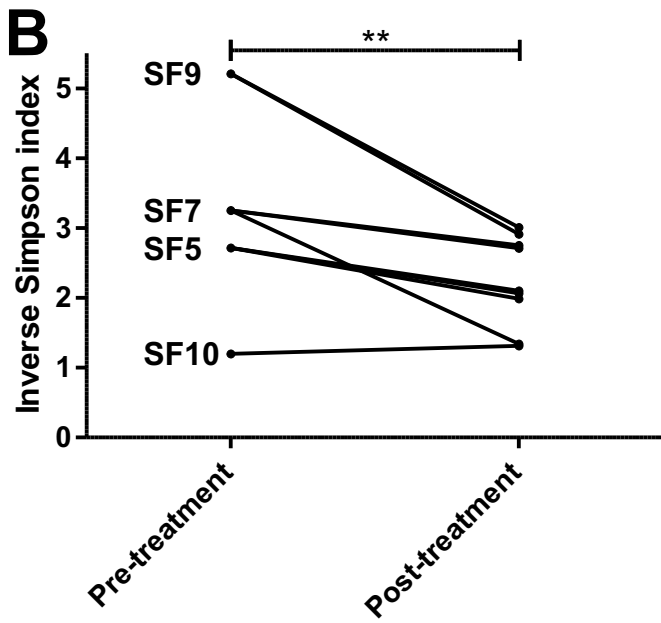

Supplement: Supplementary file 5 [file mmc5.pdf]
